# On Spontaneous Phosphorescence

**Published:** 1810-05

**Authors:** M. J. P. Dessaignes


### *On Spontaneous Phosphorescence, by* M. J. P. Dessaignes,

(From the Journal de Physique.)

I Have distinguished two sorts of spontaneous phosphorescence, the one transitory and fugitive, such as takes place on the union of a small quantity of water with quick lime; the other durable and permanent, as in luminous wood. I have spoken of the first kind formerly, and shall therefore confine myself in this place to an inquiry into the second species.

I ought in truth to acknowledge, that I have been anticipated in this part by N. Hulme, who in 1800, presented to the Royal Society of London, a very curious Memoir upon spontaneously luminous substances; but he directed his attention chiefly to fishes, and besides, notwithstanding all the interesting facts he has given us, he still left the question undecided. We shall see by what follows, that I have gone much further than he has done upon this subject, and that I have founded the explanation of this phenomenon upon the experimentum crucis of Bacon.

All wood, of whatever nature, is capable of becoming luminous, whether the trees are cut down alive, or whether they are suffered to rot upon the stumps, provided water penetrates into them, at a temperature from 8 to 12 degrees,* and they are in contact with atmospheric air. The wood of the oak turns brown, and changes its colour before it becomes phosphorescent. When the wood is compact, it is only the surface which is luminous; the inside of it is dark. Sound and dry wood is of the specific gravity 0,737; phosphorescent wood that is compact, and has not lost all its moisture 0,655; wood that has ceased to shine, and is dried by the air 0,349; it loses therefore in the act of phosphorescence more than half its original weight. The air pump has shewn me, that wood in full phosphorescence contains in its texture, more than one fourth its volume of atmospheric air.

The flesh of beef, of veal, and of fresh water fish, does not become phosphorescent so readily as sea fish. All these substances require a moderate temperature, as from 8 to 12 degrees, a constant moisture, and the contact of atmospheric air. Sea water, or a solution of salt, of equal strength, favours the developement of phosphorescence. The light with which fishes shine proceeds only from the surfaces; in-

E e 2 deed,

* Thermometre centigrade.

deed, the internal surfaces do not give out any light until they have been exposed to the air. The mucous parts, as the aponeuroses, the ligaments, the capsules, and the roes, are the most luminous.

I have examined by the eudiometer, the air contained in luminous wood: one measure of this, mixed with one of nitrous gas, produced an absorption of 0,53. A like experiment with atmospheric air, gave me 0,57. The atmospheric air therefore, which composes part of the substance of wood, is a vitiated air. I placed in an air-pump, a mass of luminous wood contained in a vessel of mercury inverted over a reservoir of the same fluid, and through the air extracted from the wood I passed lime-water, which instantly became turbid: it gives out therefore carbonic acid. Two pieces of wood taken from the same stump, the one not yet luminous, and the other in full phosphorescence, were plunged separately into two vessels filled with water; the first, after twenty days maceration, afforded only some colouring particles and extractive matter; the water of the second, nearly colourless, was a little turbid, and we could see about the wood a greyish flocculent matter, which fell to the bottom of the vessel, under the form of jelly, to give place to a further similar formation; this matter exhaled a fœtid animal odour; it appeared to me to discover the properties of vegetable albumen. I macerated in distilled water, equal quantities of the saw-dust of sound oak wood, and of wood which had ceased to shine; the water of the first was very high coloured, and gave a precipitate with glue, with sulphate of iron, and with potash; the other was insensible to these reagents, it only became a little turbid with the potash. The water in which luminous wood was macerated, was easily rendered turbid on the third day; the colourless extract which it holds in solution, is more abundant than in sound wood, and appears to be much more concrescible. This same wood decomposed by sulphuric acid, gave as its residue, a coal of a bright and velvet black, like that from burnt gum: the same wood, when in a sound state, treated in the same manner, afforded a coal of a much coarser grain, and less black. The phosphorescence ceased to show itself long before the destruction of the wood; when it had no longe the property of shining, it appeared to have lost all its physical qualities, its flexibility, its strength of texture, and a great part of its weight; but the woody structure in the parenchyma was preserved, though it was deprived of its force of cohesion, and it likewise retained all its chemical properties, for it is insoluble in water and in alcohol, and is attackable as before fore by the acids. It burns without flame, diffusing a very acrid smoke, and consumes away like tinder. It is well ascertained as to fishes, that during the whole time of their phosphorescence, they manifest no approach to putrefaction, nor is any change produced in the muscular fibre. But I have observed that the mucous juices which ooze out of the newly cut surfaces, are at first transparent and fluid; but when they become phosphorescent, they are sensibly viscid and clammy, and at last grow turbid, and there is separated from their substance a greyish powder, which gives them a sanious appearance: nevertheless, even this change is prior to their spontaneous decomposition. The muscular fibre, therefore, is not the real subject of phosphorescence, and this phenomenon has nothing in common with putrefaction.

Wood shines an equal length of time in distilled water and in river water, in each it does not give over shining till the end of six hours. Fish do not continue luminous in either of them longer than two hours. It is not true then, as Hulme affirms, that pure water extinguishes their phosphorescence immediately. Shining wood put into distilled water, and submitted to a pressure equal to 28 inches of mercury, becomes sensibly brighter under that pressure, but this luminous emanation does not continue longer than an hour.

Luminous wood and fishes continue to give out light for some time in azotic, hydrogen, and carbonic acid gases; but at length it grows weaker, and is entirely extinguished. We cannot but notice, when we are witnessing these experiments, that the phosphorescent matter, while it is shining, is already saturated with oxygen, which may serve for its combustion. Thus when we plunge them into water, and when we submit them to the air-pump, we can see a great quantity of air disengaged from both these substances. Two pieces of wood put into two vessels, one filled with oxygen gas, and the other with atmospheric air, shone an equal length of time and in a nearly similar manner. This same substance enclosed in atmospheric air, compressed by a column of mercury of 48 inches, shone in it with a much more brilliant light, and was sooner extinguished than in ordinary air. I left some luminous wood for three successive days in atmospheric air over distilled water; at the end of 12 hours there was an evident absorption equal to $\frac{1}{4}$ of a line, which went on increasing. When we enclose the air in a vessel over mercury, and let the wood remain in it, the level descends below the marked line, indicating a disengagement rather than an absorption, but when lime water is passed through it, it becomes turbid, and the volume of the water is diminished. We may observe the same phenomenon with luminous fishes, and we obtain the same results, but the absorption is more considerable.

It cannot be doubted, I think, after all the facts I have related, but that spontaneous phosphorescence is a species of combustion, in which there are formed water and carbonic acid. The substance then which affords it in wood and in fishes, must undergo a sensible change, because it loses some of its principles, and because, according to appearances, it is itself attaining a great degree of oxydation. But the ligneous principle in the wood, and the muscular fibre in the fish remain unaltered in the act of phosphorescence. In truth, the wood after this phenomenon, is clearly perceived to be altered in texture, and to have lost its most inflammable principle; but the ligneous particles still exist in the parenchyma, and appear to have retained all their physical and chemical properties. Their want of cohesion only announces the absence of a glutinous principle, which served to bind them together. This principle is to wood, what the gelatine is to the phosphate of lime in bones. Phosphorescence then, in this respect, is a phenomenon analogous to the steeping of hemp *(rouissage de chanvre)*, and to the preparation which rags undergo in the fermenting trough of the paper mill. The limits which I have prescribed myself, will not permit me to give more than the results of my observations. I shall content myself with saying, that phosphorescence in wood is produced by a glutinous extractive juice, which serves as the connecting medium of all the molecules. This juice, by reason of its union with the ligneous fecula, prevents its being attacked by water, although this may have had ready access to it; but as soon as the oxygen of the atmosphere is combined with it, some of its combustible principles being set at liberty, its attractive force for the fecula is at an end, and it becomes soluble in water. This is the reason why rotten wood affords a great deal more colourless extractive than the same wood when sound, and that this extractive is so much more concrescible, and more disposed to form pellicles or gelatinous floccules. The matter which is capable of becoming luminous in wood, absorbs oxygen, and is acted upon by all the reagents, even the weakest; and extractive is the only vegetable substance which posseses these properties in any very great degree. Phosphorescence phorescence wood is not then the first step of spontaneous decomposition, as has been thought, but is clearly the result of a transformation in the mixt body, and of its progress towards oxydation; for I have satisfied myself that the glutino-extractive, when oxygenated and dissolved in water, passes successively through the vinous, acetous, and putrid fermentations.

The muscular fibre in animals ought not any longer to be considered as the subject of phosphorescence, for it undergoes no alteration during that phenomenon, and it is ascertained that this species of combustion is anterior to putrefaction. Besides, all the colourless parts, and those which abound in mucous juices, as the skin, the ligaments, and the roes, are precisely those which are the most luminous. The matter which in animal organs has the property of shining, is the albumino-mucous juice, for that alone has the power of spontaneously absorbing oxygen from the atmosphere; and we may observe, that it is only this which discovers some change in the act of phosphorescence, for it evidently becomes more viscid and plastic, and at length becomes turbid. In a temperature below 0, the mixt body remains undisturbed, and goes on at last to putrefaction without being luminous. At a very high degree of heat the constituent principles, freed all at once from the attraction of cohesion, which was an obstacle to any new arrangement of them, become tumultuously intermingled, and endeavour to form binary compounds, according to the order of their elective attractions, and these are such as do not produce any luminous emanation. Under a moderate temperature, the primary elements being but little affected by the heat, preserve their union; the equilibrium of their affinities, although disturbed, is not entirely destroyed. In this state of effort and resistance, the absorbed oxygen detaches from the substance a portion of its hydrogen and carbon, to unite itself singly with these, and it is to this partial elimination that the phenomenon of phosphorescence is to be attributed.

Thus, instead of regarding heat as a favourable circumstance for the developement of this property, it would be more just to say that it owes its origin to a moderate degree of cold, which by virtue of its antiseptic power, retards the approach of putrefaction. Saturated saline solutions produce similar effects to cold upon the albumino-mucous juice; fresh water may in this respect be compared to heat, and very weak saline solutions to a moderate cold.

This juice, after having afforded water and carbonic

 acid, acid, and being itself furnished with a larger quantity of oxygen, (for it must be observed, that before phosphorescence, the absorbed oxygen is only solidified in the substance, and not yet combined with it) becomes insoluble and concrete. Phophorescence, therefore, is a phenomenon analagous to that operation which takes place in respiration, and in all the vascular system, by which the lymph in undergoing various changes, and in receiving new proportions of oxygen, passes successively to the states of concrescible albumen, gelatine, and fibrine. This is the grand work of living nature, to which is owing the emanations of heat, which maintain our bodies in an equal temperature, and we can but believe, that this general transformation is accompanied by a disengagement of light, that would be sensible to our sight, if the opacity of the sides of the vessels did not prevent it. The act of phosphorescence is not then the commencement of putrefaction, but is evidently a continued progress of a substance imperfectly combined to a more intimate state of combination; a state, nevertheless, in which it does not long remain in substances deprived of life, because the action of water, of air, and of temperature, soon destroys this equilibrium, which can only subsist under the influence of the vital powers.

